# Synergistic Charring Flame-Retardant Behavior of Polyimide and Melamine Polyphosphate in Glass Fiber-Reinforced Polyamide 66

**DOI:** 10.3390/polym11111851

**Published:** 2019-11-10

**Authors:** Wei Tang, Yanfang Cao, Lijun Qian, Yajun Chen, Yong Qiu, Bo Xu, Fei Xin

**Affiliations:** 1 School of Materials Science and Mechanical Engineering, Beijing Technology and Business University, Beijing 100048, China; tangwei3721@126.com (W.T.); cyfbtbu@sina.com (Y.C.); chenyajun@th.btbu.edu.cn (Y.C.); yongqiu@btbu.edu.cn (Y.Q.); xubo@btbu.edu.cn (B.X.); xinfei@th.btbu.edu.cn (F.X.); 2 Engineering Laboratory of non-halogen flame-retardants for polymers, Beijing 100048, China

**Keywords:** flame retardant, polyamide 66, polyimide, charring, synergistic effect

## Abstract

The synergistic charring, flame-retardant behavior of the macromolecular charring agents polyimide (PI) and melamine polyphosphate (MPP) were studied in glass fiber-reinforced polyamide 66 (PA66). This kind of synergistic charring effect is explained by the fact that PI performed better char-forming ability while working with phosphorus content. The research results showed that, compared with the incorporation of individual MPP, MPP/PI with an appropriate ratio exhibited better flame retardancy and better charring ability. A blend of 11.9%MPP/5.1%PI/PA66 possessed an increased LOI (limiting oxygen index) value of 33.9% and passed the UL94 V-0 rating, obtained a lower peak heat release rate value (pk-HRR), a lower total heat release (THR) value, a lower total smoke release (TSR) value, and a higher residue yield. The results verified the synergistic flame-retardant effect between MPP and PI in the PA66 composite. Melamine polyphosphate and PI jointly interacted with PA66 matrix and locked more carbonaceous compositions in residue and formed a more compact char layer, resulting in a reduced burning intensity and a reduction in the release of fuels. Therefore, the enhanced flame-retardant effect of the MPP/PI system is attributed to the higher charring ability and stronger barrier effect of the char layer in PA66 in the condensed phase.

## 1. Introduction

Polyamide 66 (PA66) is widely applied as an engineering plastic due to the fact of its excellent melt flowability, heat resistance, and mechanical properties. However, the PA66 material easily burns, because it contains a large amount of aliphatic methylene structures [1,2]. With the development of advanced manufacturing processes, higher security requirements have been applied to the flame-retardant and mechanical properties of PA66 materials in fields such as electronic appliances, automobiles, and high-speed railways [3]. In recent years, glass fiber-reinforced PA materials were introduced and used in gears, the automotive industry, and a variety of domestic appliances; glass fiber reinforcement leads to a substantial increase in the tensile strength, hardness, creep resistance, etc. [4]. However, the wick effect caused by glass fiber makes PA materials burn more easily in cases of fire [5]. Therefore, research and exploration on halogen-free flame-retardant systems for polyamide materials are particularly important for the development of high-end manufacturing of glass fiber-reinforced PA materials [6,7]. Halogen-free flame-retardants based on phosphorus, nitrogen, and other inorganic compounds are commonly used in polyamide materials. However, due to the traditional use of melamine polyphosphate (MPP) [8] and aluminum diethylphosphinate (AlPi) [9], it is difficult to meet the increasing industrial demands for their material properties, like high additive amounts, acidity, and poor compatibility. Hence, there is an urgent demand to explore more effective and stable flame-retardant systems [10,11]. Among previous studies, researchers mainly developed flame-retardant polyamide materials with high efficiency through the following three aspects. Firstly, compounding different flame-retardant components was used to research flame-retardant systems with excellent synergistic effect. Li et al. [12,13,14] combined nano-silica, silicone resin, and sepiolite with AlPi to prepare flame-retardant PA66. Krifa et al. [15] and Bourbigot et al. [16] used montmorillonite in combination with other flame-retardant components. Wang et al. used melamine cyanurate (MCA) with magnesium hydroxide and red phosphorus in PA66 materials [17,18]. Our team explored the synergistic flame-retardant effect of a phosphaphenanthrene derivative and AlPi in PA66 materials [19]. Secondly, chemically bonding different flame-retardant groups with traditional flame-retardant groups or preparing their novel derivatives are other ways to obtain flame-retardant compounds with high efficiency in polyamide. Bounor-Legare et al. and Yang et al. prepared new phosphaphenanthrene compounds [20,21]. Li et al. improved the flame-retardant performance of PA66 composites by grafting zinc to the MPP molecular structure [22]. Thirdly, designing and preparing new flame-retardant molecules with novel chemical structures or modifying the compounds which were not applied in flame-retardant fields before are also main ways to explore novel flame-retardant systems in polyamide. Wang et al. prepared novel phosphorus-containing halogen-free ionic liquids [23]. Wang et al. and Majka et al. prepared new systems based on chitosan separately [24,25]. Wang et al. synthesized a molecule containing phosphoric acid and borate groups [26]. Based on the above approaches, many researchers have conducted studies on finding high-efficiency flame-retardant systems for polyamide.

In the abovementioned flame-retardant systems, most studies indicated the importance of the charring effect in flame-retardant polyamide. Improved charring ability can lock more inflammable components, reduce the release of fuels, and provide better barrier protection for the substrate [27,28,29]. Schartel et al. coated the PA66 surface with polyimide (PI) which can promote char formation and improve the flame retardancy [30]. Piperazine pyrophosphate and linear novolac resin have also been applied in polyamide materials to improve the charring effect [31,32]. Therefore, enhancing the charring effect is an effective way to increase the working efficiency of flame-retardant systems in the polyamide.

In this article, we selected a type of PI as charring agent which possesses good compatibility because the chemical groups of PI are similar to PA66 matrix. The flame-retardant glass fiber-reinforced PA66 composites were prepared by incorporating PI and MPP. Then, the synergistic flame-retardant effect and mechanism of the MPP/PI system were investigated comprehensively.

## 2. Experimental

### 2.1. Materials

The granular polyamide 66 (EPR27) was supplied by Pingdingshan Engineering Plastics Co., Ltd., Pingdingshan, China. Short glass fiber (568H) was produced by Jushi Group Co., Ltd., Guangzhou, China. Melamine polyphosphate (MPP) was provided by Shouguang Weidong Chemical Co., Shouguang, Ltd., China. The macromolecular charring agent of polyimide (150FN019) was produced by E.I. Dupont de Nemours & Co., Ltd., Wilmington, DE, USA. Antioxidant 1010 and 168 were bought from Sinopharm Chemical Reagent Co., Ltd., Shanghai, China.

### 2.2. Preparation of Flame-Retardant PA66 Composites

All the materials were dried in a vacuum oven at 120 °C for 3.5 h. Afterwards, according to the specific ratio, all the components were melted and blended using torque rheometer (XSS-300, Shanghai Kechuang Rubber and Plastic Machinery Co., Ltd., Shanghai, China.) at 270 °C for 10 min. One hundred and eighty grams of composite materials were obtained each time. Then, the acquired composites were compressed to thick standard specimens at 270 °C under 35 MPa using the tablet machine. The detailed formulae of the flame-retardant PA66 composites are listed in Table 1. Both MPP and PI were used as flame-retardants, and the glass fiber (GF) was used as reinforcement. In addition, the total amount of antioxidants in all samples was 0.6 wt %, and the mass ratio of antioxidant 1010 to antioxidant 168 was 2:1.

### 2.3. Characterization

The limiting oxygen index (LOI) values were measured on an FTT (Fire Testing Technology, London, UK) Dynisco LOI instrument according to ASTMD 2863-97 with a sample dimension of 100.0 × 6.5 × 3.2 mm^3^. The vertical burning test (UL94) for combustion level was performed using an FTT0082 instrument (Fire Testing Technology, London, UK) according to ASTMD 3801 with sample dimension of 125.0 × 12.7 × 3.2 mm^3^.

Fire behavior was characterized on an FTT cone calorimeter (Fire Testing Technology, London, UK)) according to ISO5660 under a 50 kW/m^2^ external heat flux with a sample dimension of 100.0 × 100.0 × 3.2 mm^3^. Time to ignition (TTI), total heat release (THR), total smoke release (TSR), and other typical parameters were collected and recorded synchronously. The results were the mean values from two measurements.

The thermal gravimetric analysis (TGA) was performed using a Perkin–Elmer instrument STA 8000 (Perkin Elmer, Waltham, MA. USA) thermal gravimetric analyzer. The sample was placed in a zirconia crucible and heated from 50 °C to 700 ° C at the rate of 20 °C/min in N_2_ atmosphere. All tests were repeated two times.

The microscopic morphology of the residual char after the cone calorimeter test was observed at a magnification of 800× according to a Phenom Pro and TESCAN VEGA II scanning electron microscopes (SEM) under vacuum conditions with a voltage of 10 kV. The energy-dispersive X-ray spectrometer (EDX, Phenom World, Eindhoven, Netherlands) of residual char after the cone calorimeter test was performed at a magnification of 800× under vacuum and high current conditions with a voltage of 15 kV.

The Fourier transform infrared (FTIR) spectra were obtained with a Nicolet iN10MX-type spectrometer (Thermo Nicolet Corp., Madison, WI, USA). The test samples were the residues from UL94 test which were artificially extinguished after 10 s ignition time.

## 3. Results and Discussion

### 3.1. LOI and UL94 Tests

The LOI and UL94 tests play primary roles in exploring flame retardancy and the screening formulae of polymer samples. The results of this study are illustrated in Table 2. The LOI values of composites 11.9%MPP/5.1%PI/PA66 and 10.2%MPP/6.8%PI/PA66 were 33.9% and 33.7%, respectively, which were higher than the LOI values of the composites containing MPP or PI individually. Similarly, the 11.9%MPP/5.1%PI/PA66 possessed better flame-retardant classification, a UL94 V-0 rating, whereas the UL94 rating of 17.0%MPP/PA66 and 5.1%PI/PA66 were just a V-1 rating and no rating, respectively. The results imply that the MPP/PI mixture exhibited a better flame-retardant effect than MPP or PI when it was individually applied in the PA66 composites. The higher content of PI in PA66 would have led to a high viscosity, so the PA66 composites in the case of higher PI loadings were not studied. Besides, 10.2%MPP/6.8%PI/PA66 only reached a UL94 V-1 rating. Hence, the appropriate MPP/PI ratio can exert synergistic flame-retardant effects in the application of PA66 composites. Compared with MPP/PA66 and PI/PA66 composites, MPP/PI/PA66 had a better flame-retardant performance which indicated that PI forms char layers easier in the presence of phosphorus (MPP) compared with the PI/PA66 sample. The working mechanism of the MPP/PI system is analyzed in the subsequent discussion.

**Figure 1:**
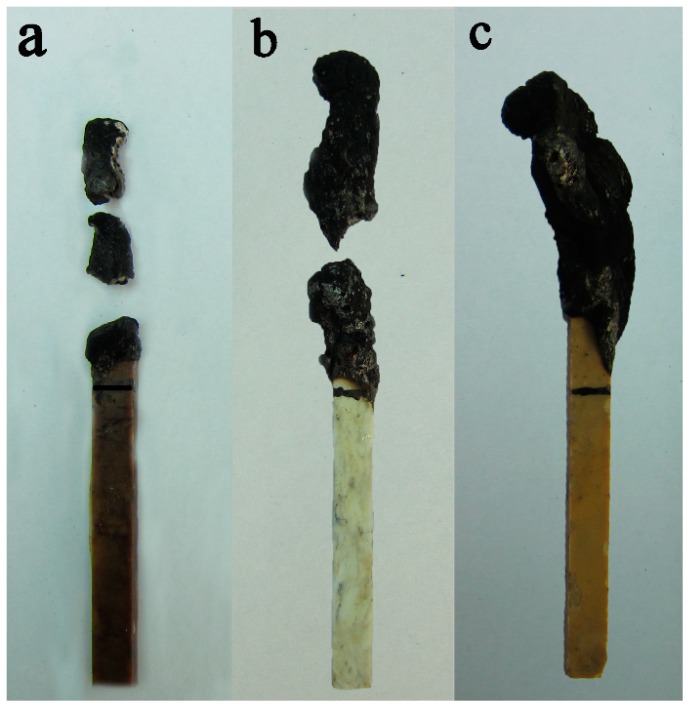
Digital photos of residues from the LOI test: (**a**) 5.1% PI/PA66; (**b**) 17.0%MPP/PA66; (**c**) 11.9%MPP/5.1%PI/PA66.

Figure 1 shows the digital photos of residues from the LOI burning test. According to Figure 1b, during the combustion of sample 17.0%MPP/PA66, although the expansive char layers were formed, the residues fell off during combustion which indicated that the intensity of the residue was weak and led the specimen to continue burning, obtaining the corresponding lower LOI value. As shown in Figure 1a, the sample 5.1%PI/PA66 showed a similar phenomenon as 17.0%MPP/PA66 and was accompanied by a large amount of residue. However, sample 11.9%MPP/5.1%PI/PA66 produced a larger residue crown with a certain intensity after the LOI test, shown in Figure 1c, which indicates that PI and MPP can work together to produce a better charring effect. Therefore, the optimized MPP/PI system facilitated the PA66 composite to form an integral and firm char layer, which contributed to the effective coverage on the matrix surface and further hindered the exchange of heat and flammable gas [33]. The residue morphology after the LOI test preliminarily disclosed the synergistic charring flame-retardant behavior of MPP/PI in PA66 composite.

### 3.2. Cone Calorimeter Test

The cone calorimeter test was also used to investigate the combustion behavior of PA66 composites and initially analyze the flame-retardant mechanism of the MPP/PI system. Several typical parameters, such as total heat release (THR), peak value of heat release rate (pk-HRR), char yield after combustion (residue), total smoke release (TSR), and average effective of heat of combustion (av-EHC), are listed in Table 3. It should be noted that the mass of 30% glass fiber was subtracted from the calculation of the residue.

**Figure 2:**
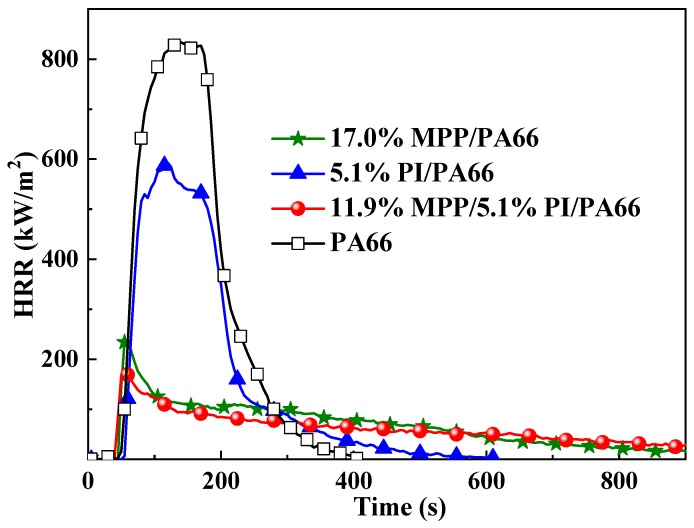
The HRR curves of the flame-retardant PA66 composites.

According to the data in Table 3, the pk-HRR value of 11.9%MPP/5.1%PI/PA66 was only 190 kW/m^2^ which is less than those of PA66 (834 kW/m^2^) and 5.1%PI/PA66 (559 kW/m^2^). Besides, the HRR value of 11.9%MPP/5.1%PI/PA66 was suppressed at a very low level during the entire burning process as shown in Figure 2. Compared with the addition of PI or MPP alone, the PI/MPP system can inhibit the combustion intensity of PA66 to a greater extent. These phenomena indicate that MPP and PI can work together to inhibit the burning intensity and improve the flame-retardant properties of PA66 composites. Moreover, the results further verified the synergistic effect between MPP and PI in the PA66 composite.

**Figure 3:**
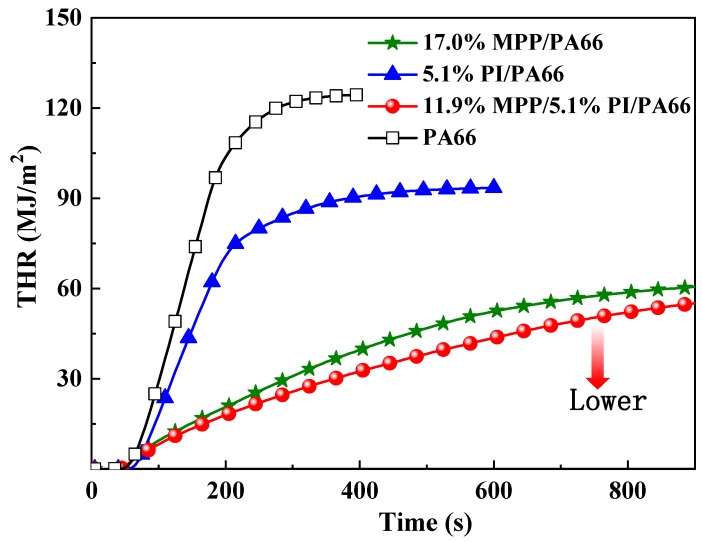
The THR curves of the flame-retardant PA66 composites.

**Figure 4:**
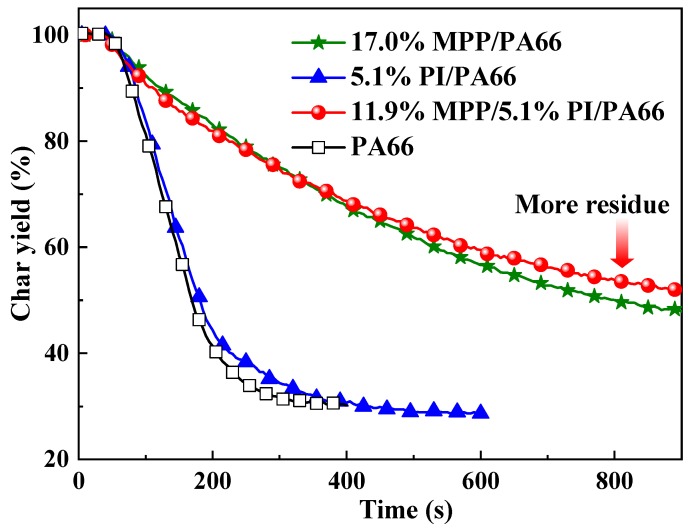
The mass loss curves of the flame-retardant PA66 composites.

The THR curves of these composites are illustrated in Figure 3 and their THR values are listed in Table 3. After ignition, the THR curve of 11.9%MPP/5.1%PI/PA66 exhibited the slowest growth rate of all the samples, which indicates that 11.9%MPP/5.1%PI/PA66 performed at a slower burning rate than the other samples and further proved that the inflammable components were difficult to release during combustion. In addition, compared with PA66 and 17.0%MPP/PA66, the THR value of 11.9%MPP/5.1%PI/PA66 was reduced by 56.5% and 9.0%, respectively. The results indicate that the MPP/PI system had a better flame-retardant effect on reducing the heat release. Usually, the decrease in THR is due to the fact of two reasons: (1) incomplete burning of inflammable components in the gas phase and (2) reduction of fuel released from the matrix. The av-EHC values of 17.0%MPP/PA66 and 11.9%MPP/5.1%PI/PA66 were approximately 24–25 MJ/kg while that of PA66 was approximately 29.5 MJ/kg. This result was mainly due to the increased char yield of 17.0%MPP/PA66 and 11.9%MPP/5.1%PI/PA66 thus locking more fuel in the condensed phase. In addition, the phosphorous substances produced by MPP may have a certain impact on the reduction of av-EHC. Besides, in comparison with the av-EHC value of 17.0%MPP/PA66, that of 11.9%MPP/5.1%PI/PA66 did not obviously change which implies that the MPP/PI system did not generate a stronger action in the gas phase than MPP. The reason for the low THR value of 11.9%MPP/5.1%PI/PA66 can be deduced from the fact that more components remained in the condensed phase. Moreover, the residue yields can disclose the charring ability and the effect of the release of reduced fuels during combustion. From the results in Table 3 and Figure 4, the composites containing 17 wt % MPP generated 18.6% residue, implying an outstanding condensed phase flame-retardant effect from MPP. However, after replacing 5.1 wt % MPP with PI, 11.9%MPP/5.1%PI/PA66 possessed the lowest total mass loss (TML) and the higher residue yields of 21.0%, indicating that the MPP/PI system further facilitated the increased residue production and reduced the release of fuels from the composite. Therefore, the reduction in the THR value of 11.9%MPP/5.1%PI/PA66 was attributed to the reduction of fuels, which was caused by a better charring effect from the MPP/PI system.

Furthermore, the higher residue yields of the MPP/PI system not only the reduced fuel release but also produced a barrier effect between the atmosphere and the underlying matrix in the condensed phase which led to the lower pk-HRR value of the MPP/PI/PA66 composite. According to the av-EHC values, the addition of PI in MPP/PA66 did not affect the value of the av-EHC composite compared with the MPP/PA66 composite. Therefore, the MPP/PI system reserved the flame-retardant effect of MPP and also strengthened the charring ability and barrier effect in the condensed phase, resulting in the enhanced flame-retardant effect on the PA66 matrix.

The production of smoke was the main hazard in the fire disaster and the value of TSR was the key secondary fire performance parameter. In contrast to TSR value of the PA66 composite without flame-retardant, that of the 11.9%MPP/5.1%PI/PA66 was reduced by 72.5%; compared with the TSR value of 17.0%MPP/PA66, that of the 11.9%MPP/5.1%PI/PA66 also decreased by 17.3%. Less smoke release from the 11.9%MPP/5.1%PI/PA66 sample decreased the fire risk significantly. The results further verified that MPP and PI had a stronger effect on locking carbonaceous components in the char layer and inhibiting the release of smoke. The reduction of smoke production was not only because a fraction of the carbon was trapped in the condensed phase but also because the decomposition rate was slowed down.

### 3.3. Thermal Properties of Flame-Retardant PA66 Composites

**Figure 5:**
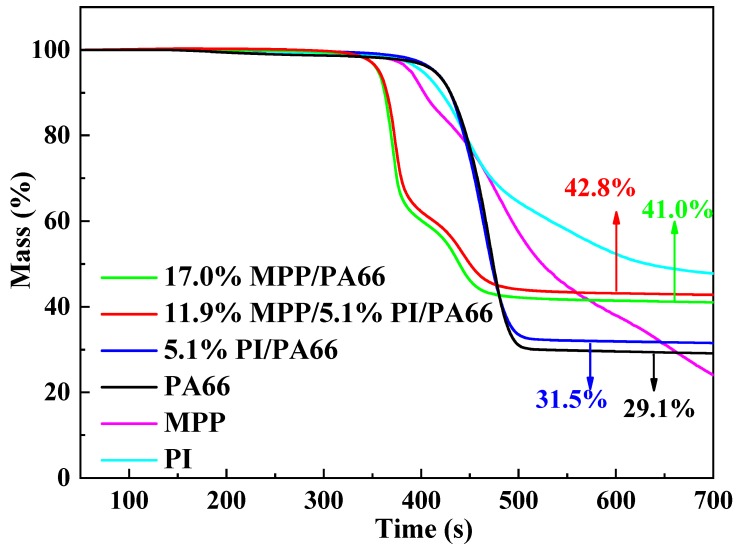
The TGA curves of the PA66 composites and flame-retardants.

The TGA curves of the PA66 composites and flame-retardants are illustrated in Figure 5. As shown in Figure 5, the flame-retardants, MPP, and PI all had a slightly lower onset degradation temperature than the neat PA66 composite. The onset degradation temperature of the 5.1%PI/PA66 composite was the same as that of PA66, which indicated that the addition of PI hardly caused the decomposition of the PA66 matrix in advance. Therefore, it can be deduced that PI hardly interacted with PA66 in the 5.1%PI/PA66 composites. However, the onset degradation temperature of 17.0%MPP/PA66 showed a decrease of nearly 60 °C compared with PA66, and the residue yield of 17.0%MPP/PA66 increased from 29.1% of PA66 to 41.0%, which implied that MPP can interact with PA66 matrix to lead the earlier decomposition of composites and higher residue yield when heated. Although PI did not affect the onset decomposition temperature of PA66, MPP in the MPP/PI system dominated the early degradation of PA66. Moreover, the MPP/PI system can further increase the charring yields of PA66 composites compared with single component MPP. The residue yields of 11.9%MPP/5.1%PI/PA66 enhanced to 42.8% from 41.0% for 17.0%MPP/PA66. This may be due to the better char-forming ability of PI. The result of the TGA test was consistent with that of the char yield in the cone calorimeter test.

### 3.4. SEM/EDX Analysis of Residue

**Figure 6:**
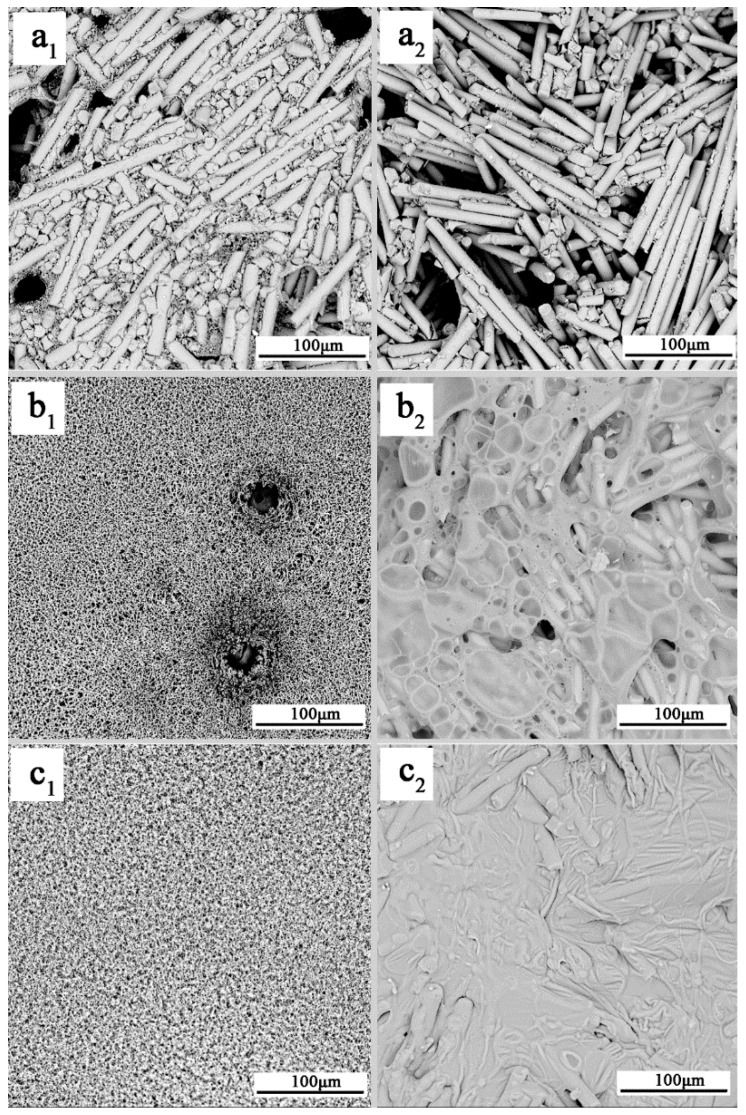
SEM photos of the top-surface (**a1**, **b1**, **c1**) and middle-tier (**a2**, **b2**, **c2**) residues from the cone calorimeter: (**a**) 5.1%PI/PA66, (**b**) 17.0%MPP/PA66, (**c**) 11.9%MPP/5.1%PI/PA66.

SEM/EDX is a powerful tool to characterize complex fire residues, because it can combine surface structure with element contents [34,35]. The SEM photos of the top surface and middle tier of residue after the cone calorimeter test are exhibited in Figure 6. The element weight percentages from the middle tier EDX tests are listed in Table 5.

The residue was composed of multi-layer structures after combustion of flame-retardant PA66. Therefore, in order to comprehensively explain the flame-retardant effect of the MPP/PI system in PA66 composites during combustion in the condensed phase, we observed the top surface and middle tier of the residues. In Figure 6(a1,2), the residue of 5.1%PI/PA66 showed the accumulation morphology of glass fiber and the existence of no carbonaceous residue, thereby proving the worse charring effect and worse barrier effect from PI during combustion. As mentioned in a previous report [36], the residue morphology of 5.1%PI/PA66 was similar to that of the neat PA66 composite without charring effect. Figure 6(b1,2) shows the carbonaceous residues covered on the outer surface and combined with glass fiber in the middle tier of the residue, although some holes and cracks still appear, implying that the MPP improved the charring effect effectively. The residue microscopic morphology of the PA66 composite with MPP/PI is shown in Figure 6(c1,2), the char layer was denser and intact, and the more carbonaceous contents combined with glass fiber and no glass fiber were exposed, testifying that the MPP/PI system promoted a more whole and more residual char layer than PI or MPP did alone. The results further demonstrated the synergistic charring effect of the MPP/PI system for PA66 composites in the condensed phase.

According to the EDX data in Table 4, compared with the results of sample 17%MPP/PA66, the carbon and nitrogen element weight percentages of 11.9%MPP/5.1%PI/PA66 both increased, which corresponded to the results of the more reserved residues during combustion. This result also implied that MPP interacted with PI and the PA66 matrix to lock more carbonaceous components in residue, resulting in a reduction in the release of fuels. Besides, the detected phosphorus content in the 11.9%MPP/5.1%PI/PA66 residue was reduced. The quantitative calculation of P was analyzed for this reason (Table 5). According to a 20.4% P content in MPP and a weight of 40 g for each sample in the cone calorimeter test, the P content of each composite was calculated: 1.38 g in 17%MPP/PA66 and 0.97 g in 11.9%MPP/5.1%PI/PA66. Then, the mass of P in residue was also calculated according to the EDX data and the residue yield: 0.48 g in 17%MPP/PA66 and 0.13 g in 11.9%MPP/5.1%PI/PA66. Therefore, during combustion, 17%MPP/PA66 released 0.9 g P and 11.9%MPP/5.1%/PA66 released 0.84 g P. Therefore, 17%MPP/PA66 released more P-containing components than 11.9%MPP/5.1%/PA66. As for Si, Ca, and Al, MPP and PI worked together to improve the dense structure of the char layers, which caused more glass fibers to be covered in residue as shown in Figure 6(c2). Therefore, lower Si, Ca, and Al contents of MPP/PI/PA66 were detected when scanning the surface elements of the residue by EDX. Therefore, during combustion, PI, MPP, and PA66 inevitably produced some reactions, which promoted PA66 composites to form a stronger charring effect in the condensed phase. 

### 3.5. FTIR Analysis of Residues

**Figure 7:**
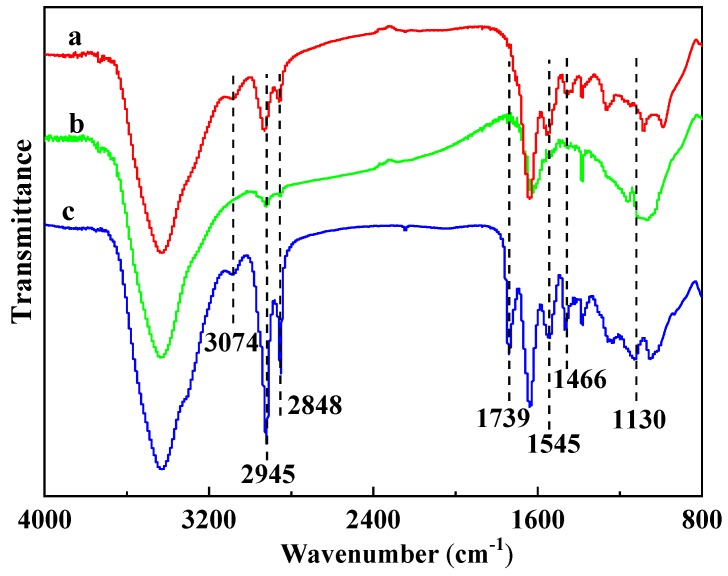
FTIR curves of the residues from the UL94 test: (**a**) 5.1%PI/PA66, (**b**) 17.0%MPP/PA66, (**c**) 11.9%MPP/5.1%PI/PA66.

The FTIR spectra of the 17.0%MPP/PA66, 5.1%PI/PA66, and 11.9%MPP/5.1%PI/PA66 residues are shown in Figure 7 to further explore the composition change of residues after the UL94 test. The absorption peaks of all the residues were similar. However, there were still some tiny differences in those spectra. Two peaks at 2945 and 2848 cm^−1^ were found in all the spectra which is typical for absorption of C–H. The strengthened C–H peaks of 11.9%MPP/5.1%PI/PA66 confirmed that more aliphatic carbonaceous contents in the PA66 matrix were locked in the residue. The peak at 1130 cm^−1^ was attributed to the P–O–C or P–O–Ph group [34] which is produced from flame-retardant MPP. Meanwhile, the appearance of the peak at positions 3074 cm^−1^, 1545 cm^−1^, and 1466 cm^−1^ indicated the existence of an aromatic ring generated from the PI and PA66 matrix. The peak at 1739 cm^−1^ was the C=O group combining with the benzene ring which was produced from the PI and PA66 matrix. The peak around 3400 cm^−1^ was attributed by the –NH– groups generated by the PA66 matrix [36]. Therefore, complex interactions occurred between MPP, PI, and PA66 during charring process. The phosphorus, more aromatic and more carbonaceous contents jointly generated more compact residue through the interactions between MPP, PI, and PA66, causing the synergistic charring, flame-retardant effect of the MPP/PI system.

## 4. Conclusions

Macromolecular charring agents, PI and MPP, were applied in glass fiber-reinforced polyamide 66 to explore more effective flame-retardant systems. The PA66 composite with a 11.9 wt % MPP and 5.1 wt % PI system increased the LOI value to 33.5%, passed the UL94 V-0 rating, suppressed the combustion intensity, reduced the total heat release, and increased the residue yields compared with the composite containing only 17wt % MPP. The results indicate a synergistic flame-retardant effect between MPP and PI in PA66 composites. The improved flame retardancy of the MPP/PI system is attributed to the synergistic charring ability from MPP/PI in PA66 during combustion. PI forms char more easily in the presence of P-contained components (MPPs) compared to the PI/PA66 sample. The MPP/PI system in the PA66 composites, during combustion, locked more carbonaceous contents and formed more compacts and more residues, resulting in reducing burning intensity and lower fuels release. The high efficiency of the MMP/PI mixture is due to the increased formation of a dense and stable carbonaceous residue. Accordingly, the MPP/PI system provides enhanced flame retardancy for PA66 composites.

## Figures and Tables

**Table 1:** Formulae of flame-retardant PA66 composites.

Samples	PA66 (wt %)	Glass Fiber (wt %)	MPP (wt %)	PI (wt %)	Antioxidant (wt %)
PA66	69.4	30	-	-	0.6
17.0%MPP /PA66	52.4	30	17.0	-	0.6
5.1%PI/PA66	64.3	30	-	5.1	0.6
11.9%MPP/5.1%PI/PA66	52.4	30	11.9	5.1	0.6
10.2%MPP/6.8%PI/PA66	52.4	30	10.2	6.8	0.6

**Table 2:** The LOI value and UL94 rating of flame-retardant PA66.

Samples	LOI (%)	UL94			
		av-t1(s)	av-t2(s)	Dripping	Rating
PA66	23.6	185.0	-	Yes	No rating
17.0%MPP/PA66	31.7	3.6	8.8	No	V-1
5.1%PI/PA66	26.3	24.6 ^a^	-	Yes	No rating
11.9%MPP/5.1%PI/PA66	33.9	2.2	3.7	No	V-0
10.2%MPP/6.8%PI/PA66	33.7	8.7	14.4	No	V-1

^a^ Time of burned to clamp.

**Table 3:** Cone calorimeter data of the flame-retardant PA66 composites.

Samples	pk-HRR (kW/m^2^)	THR (MJ/m^2^)	av-EHC (MJ/kg)	Char (wt %)	TSR (m^2^/m^2^)
PA66	830 ± 42	122.4 ± 3.7	29.5 ± 1.7	0.1 ± 1.0	3904 ± 158
17.0%MPP /PA66	201 ± 35	60.3 ± 0.2	24.4 ± 1.3	18.6 ± 0.7	1365 ± 101
5.1%PI/PA66	559 ± 33	92.8 ± 0.6	30.3 ± 0.2	0.1 ± 0.5	1420 ± 20
11.9%MPP/5.1%PI/PA66	190 ± 10	54.1 ± 1.0	24.9 ± 0.3	21.0 ± 0.9	1129 ± 96

**Table 4:** The element weight percentages of middle-tier residue in EDX.

Samples	C (%)	O (%)	N (%)	P (%)	Si (%)	Ca (%)	Al (%)
17.0%MPP/PA66	46.58	26.19	11.70	6.58	4.45	3.31	1.20
11.9%MPP/5.1%PI/PA66	69.82	9.19	18.83	1.59	0.57	0	0

**Table 5:** The P content data by calculation.

Samples	P in Composites (g)	P in Residue (g)	P Released (g)
17.0%MPP/PA66	1.38	0.48	0.90
11.9%MPP/5.1%PI/PA66	0.97	0.13	0.84
